# Supplementary material for: The Hox transcription factor Ubx stabilizes lineage commitment by suppressing cellular plasticity in Drosophila
Source: eLife. 2019 May 3;8:e42675. doi: 10.7554/eLife.42675 (PMC6513553; doi:10.7554/eLife.42675)
Supplement: Supplementary file 1. [file elife-42675-supp1.docx]

| Gene set | Gene | Literature |
| --- | --- | --- |
| mesodermal lineage  stage 10-13  RNA-Seq | *tin* | Azpiazu and Frasch, 1993 |
|  | *Mef2* | Bour et al., 1995 |
|  | *org-1* | Schaub et al., 2012 |
|  | *bin* | Zaffran et al., 2001 |
|  | *mib2* | Nguyen et al., 2007 |
| mesodermal lineage  stage 14-17  RNA-Seq | *tn* | Domsch et al., 2013 |
|  | *Actn* | Kreisköther et al., 2006 |
|  | *Tm1* | Gremke et al., 1993 |
|  | *Mef2* | Lilly et al., 1995*;* Bour et al., 1995 |
|  | *Zormin* | Kreisköther et al., 2006 |
| neuronal lineage  stage 10-13  RNA-Seq | *dpn* | Bier et al., 1992 |
|  | *Jra* | Bossing et al., 2012 |
|  | *NT1* | Zhu et al., 2008 |
|  | *tsh* | McCormick et al., 1995 |
| neuronal lineage  stage 14-17  RNA-Seq | *Fas2* | Grenningloh et al., 1991 |
|  | *Syt4* | Littleton et al., 1993 |
|  | *Nrg* | Bieber et al., 1989 |
|  | *Nct* | López-Schier and St Johnston, 2002 |
|  | *ncd* | Sharp et al., 2000 |

**Supplementary File 1. Table of known genes expressed in the mesodermal or neuronal lineages.**

References

Azpiazu, N., and Frasch, M. (1993). tinman and bagpipe: two homeo box genes that determine cell fates in the dorsal mesoderm of Drosophila. Genes Dev. *7*, 1325–1340.

Bieber, A.J., Snow, P.M., Hortsch, M., Patel, N.H., Jacobs, J.R., Traquina, Z.R., Schilling, J., and Goodman, C.S. (1989). Drosophila neuroglian: a member of the immunoglobulin superfamily with extensive homology to the vertebrate neural adhesion molecule L1. Cell *59*, 447–460.

Bier, E., Vaessin, H., Younger-Shepherd, S., Jan, L.Y., and Jan, Y.N. (1992). deadpan, an essential pan-neural gene in Drosophila, encodes a helix-loop-helix protein similar to the hairy gene product. Genes Dev. *6*, 2137–2151.

Bossing, T., Barros, C.S., Fischer, B., Russell, S., and Shepherd, D. (2012). Disruption of microtubule integrity initiates mitosis during CNS repair. Dev. Cell *23*, 433–440.

Bour, B.A., O'Brien, M.A., Lockwood, W.L., Goldstein, E.S., Bodmer, R., Taghert, P.H., ABMAYR, S.M., and NGUYEN, H.T. (1995). Drosophila MEF2, a transcription factor that is essential for myogenesis. Genes Dev. *9*, 730–741.

Domsch, K., Ezzeddine, N., and Nguyen, H.T. (2013). Abba is an essential TRIM/RBCC protein to maintain the integrity of sarcomeric cytoarchitecture. J. Cell. Sci. *126*, 3314–3323.

Gremke, L., Lord, P.C., Sabacan, L., Lin, S.C., Wohlwill, A., and Storti, R.V. (1993). Coordinate regulation of Drosophila tropomyosin gene expression is controlled by multiple muscle-type-specific positive and negative enhancer elements. Dev. Biol. *159*, 513–527.

Grenningloh, G., Rehm, E.J., and Goodman, C.S. (1991). Genetic analysis of growth cone guidance in Drosophila: fasciclin II functions as a neuronal recognition molecule. Cell *67*, 45–57.

Kreisköther, N., Reichert, N., Buttgereit, D., Hertenstein, A., Fischbach, K.-F., and Renkawitz-Pohl, R. (2006). Drosophila rolling pebbles colocalises and putatively interacts with alpha-Actinin and the Sls isoform Zormin in the Z-discs of the sarcomere and with Dumbfounded/Kirre, alpha-Actinin and Zormin in the terminal Z-discs. J. Muscle Res. Cell. Motil. *27*, 93–106.

Lilly, B., Zhao, B., Ranganayakulu, G., Paterson, B.M., Schulz, R.A., and Olson, E.N. (1995). Requirement of MADS domain transcription factor D-MEF2 for muscle formation in Drosophila. Science *267*, 688–693.

Littleton, J.T., Bellen, H.J., and Perin, M.S. (1993). Expression of synaptotagmin in Drosophila reveals transport and localization of synaptic vesicles to the synapse. Development *118*, 1077–1088.

López-Schier, H., and St Johnston, D. (2002). Drosophila nicastrin is essential for the intramembranous cleavage of notch. Dev. Cell *2*, 79–89.

McCormick, A., Coré, N., Kerridge, S., and Scott, M.P. (1995). Homeotic response elements are tightly linked to tissue-specific elements in a transcriptional enhancer of the teashirt gene. Development *121*, 2799–2812.

Nguyen, H.T., Voza, F., Ezzeddine, N., and Frasch, M. (2007). Drosophila mind bomb2 is required for maintaining muscle integrity and survival. J. Cell Biol. *179*, 219–227.

Schaub, C., Nagaso, H., Jin, H., and Frasch, M. (2012). Org-1, the Drosophila ortholog of Tbx1, is a direct activator of known identity genes during muscle specification. Development *139*, 1001–1012.

Sharp, D.J., Rogers, G.C., and Scholey, J.M. (2000). Cytoplasmic dynein is required for poleward chromosome movement during mitosis in Drosophila embryos. Nat. Cell Biol. *2*, 922–930.

Zaffran, S., Küchler, A., Lee, H.H., and Frasch, M. (2001). biniou (FoxF), a central component in a regulatory network controlling visceral mesoderm development and midgut morphogenesis in Drosophila. Genes Dev. *15*, 2900–2915.

Zhu, B., Pennack, J.A., McQuilton, P., Forero, M.G., Mizuguchi, K., Sutcliffe, B., Gu, C.-J., Fenton, J.C., and Hidalgo, A. (2008). Drosophila neurotrophins reveal a common mechanism for nervous system formation. PLoS Biol. *6*, e284.
