# Supplementary material for: The Hox transcription factor Ubx stabilizes lineage commitment by suppressing cellular plasticity in Drosophila
Source: eLife. 2019 May 3;8:e42675. doi: 10.7554/eLife.42675 (PMC6513553; doi:10.7554/eLife.42675)
Supplement: Supplementary file 2. [file elife-42675-supp2.docx]

| gene | name | Sequence |
| --- | --- | --- |
| *RpL32* | RpL-RT-For | TAC AGG CCC AAG ATC GTG AA |
|  | RpL-RT-Rev | TCT CCT TGC GCT TCT TGG A |
| *Mef2* | Mef2-RT-For | GCC AGG TGC GAG AGA CTA GA |
|  | Mef2-RT-Rev | ATC AAC GAC AGA GCC AGA CA |
| *nau* | nau-RT-For | AGA TCC TCC AGG CTC TCG AT |
|  | nau-RT-Rev | GCA AAA AGA AGA GCG TCA CC |
| *en* | en-RT-For | CGT CGT TGG TCT TGT CCT TT |
|  | en-RT-Rev | GGA ATG GAG TCC TCG GAT G |
| *elav* | elav-RT-For | CAA TAC GAA TGG CAA TGC AG |
|  | elav-RT-Rev | TTG TCG CGT ATC AGC TTC AC |
| *eg* | eg-RT-For | CGG TCC AAC TGG TTC AAG AT |
|  | eg-RT-Rev | GAC TTG ATG CAG GGA TTG GT |
| *pho* | pho-qPCR-For3 | ACA TTC GAG GGA TGC GGA AA |
|  | pho-qPCR-Rev3 | CCA CTT TTC CCC GAA ATA CTG G |

**Supplementary File 2. Primers used for qPCR experiments to test total RNA.**
