# Supplementary material for: The Hox transcription factor Ubx stabilizes lineage commitment by suppressing cellular plasticity in Drosophila
Source: eLife. 2019 May 3;8:e42675. doi: 10.7554/eLife.42675 (PMC6513553; doi:10.7554/eLife.42675)
Supplement: Supplementary file 3. [file elife-42675-supp3.docx]

| gene | name | Sequence |
| --- | --- | --- |
| *vnd* | vnd-qPCR-For2 | TAA CGC AGA GTC CCC AAA GC |
|  | vnd-qPCR-Rev2 | CCA TCG TCG CGG ACA TGT |
| *HGTX* | HGTX-For | GCT GCT TTT GTG GCT GCT TT |
|  | HGTX-Rev | GCA GCA GCA GCA GGA TAA TT |
| *ham* (Ubx) | ham-me-qPCR-For1 | GTC CGA AAC CGA ATA GAG TCG A |
|  | ham-me-qPCR-Rev1 | GTT AAG GGG CGG GGG ATA TG |
| *ham* (Pho) | ham-me3-qPCR-For1 | CCG TCT TTC CGG CAC AAA AT |
|  | ham-me3-qPCR-Rev1 | TTT CTT GTC GCC TCG ATT GC |
| *Ptx-1* (Ubx) | Ptx1-me3-qPCR-For | GAT TCC GGA ATG GGG GTT GT |
|  | Ptx1-me3-qPCR-Rev | TTG CCA CAA AAC CGA CAC AC |
| *Ptx-1* (Pho) | Ptx1-me2-qPCR-For | AAT GTC CCA TCT GCA GGT CG |
|  | Ptx1-me2-qPCR-Rev | GAC CTT CTT CTT GTC GGC CA |
| *NT1* (Ubx) | NT1_2_For | CCC AAG GAG CCG ACA AAT CT |
|  | NT1_2_For | AAC TGT CAA CTG TTT GCC GC |
| *NT1* (Pho) | NT1-me6-qPCR-For | ATT ACG ACC GTG CAG CAA GA |
|  | NT1-me6-qPCR-Rev | GCA ACA ACC AGA GCA GCT TC |
| *scramb1* (control1) | Scramb-For | GAG CGA AAA GGT GCA GAA AG |
|  | Scramb-Rev | CCT GTC TGT TTC CCT GCT GT |

**Supplementary File 3. Primers used for qPCR to test genomic loci identified by ChIP experiments.**
