# Supplementary material for: The Hox transcription factor Ubx stabilizes lineage commitment by suppressing cellular plasticity in Drosophila
Source: eLife. 2019 May 3;8:e42675. doi: 10.7554/eLife.42675 (PMC6513553; doi:10.7554/eLife.42675)
Supplement: Supplementary file 4. [file elife-42675-supp4.docx]

| mesoderm | neuronal | ectoderm | gut & endoderm |
| --- | --- | --- | --- |
| GO:0008078 | GO:0050905 | GO:0060802 | GO:0060423 |
| GO:0061061 | GO:0022008 | GO:0006509 | GO:0035069 |
| GO:0007498 | GO:0097187 | GO:0071696 | GO:0003133 |
| GO:0090078 | GO:0061837 | GO:0007499 | GO:0007492 |
| GO:0035994 | GO:0060234 | GO:0007439 | GO:0007374 |
| GO:0033002 | GO:0021675 | GO:0007398 | GO:0007375 |
| GO:0060537 | GO:0097402 | GO:1905327 | GO:0072002 |
| GO:0110021 | GO:0007129 | GO:0060438 | GO:0007431 |
| GO:0099622 | GO:0007417 | GO:1905177 | GO:0055123 |
| GO:0061026 | GO:0045213 | GO:0042335 | GO:0007586 |
| GO:0043403 | GO:0038189 | GO:0007424 | GO:0002387 |
| GO:0014719 | GO:0038179 | GO:0048104 | GO:0048537 |
| GO:0010657 | GO:0070050 | GO:0048104 | GO:1902900 |
| GO:1902766 | GO:0001505 | GO:0048736 | GO:0016477 |
| GO:0014812 | GO:0023041 | GO:0046843 |  |
| GO:0014870 | GO:0060052 | GO:0097374 |  |
| GO:0014850 | GO:0060563 | GO:1904937 |  |
| GO:2000035 | GO:0110088 | GO:0048880 |  |
| GO:0086001 | GO:0006836 | GO:0007600 |  |
| GO:0003012 | GO:0099628 | GO:0007423 |  |
| GO:0046716 | GO:0099637 | GO:0042303 |  |
| GO:0007495 | GO:0099590 |  |  |
| GO:1990764 | GO:0099538 |  |  |
| GO:0086036 | GO:0106027 |  |  |
| GO:1904516 | GO:0070997 |  |  |
| GO:0036446 | GO:0014730 |  |  |
| GO:1905285 | GO:0010813 |  |  |
| GO:0035989 | GO:0014852 |  |  |
| GO:1905072 | GO:0048884 |  |  |
| GO:0061384 | GO:0048883 |  |  |
| GO:0003015 | GO:1902988 |  |  |
| GO:0007507 | GO:0061532 |  |  |
| GO:1905223 | GO:0036520 |  |  |
| GO:0044556 | GO:0098873 |  |  |
| GO:0140199 | GO:0097719 |  |  |
| GO:0140193 | GO:0048702 |  |  |
| GO:0140194 | GO:0021995 |  |  |
| GO:0140195 | GO:0070657 |  |  |
| GO:0140196 | GO:0032898 |  |  |
| GO:0140192 | GO:0036445 |  |  |
| GO:0140200 | GO:0007218 |  |  |
|  | GO:0007158 |  |  |
|  | GO:0035860 |  |  |
|  | GO:0031644 |  |  |
|  | GO:0070120 |  |  |
|  | GO:0099011 |  |  |
|  | GO:0099014 |  |  |
|  | GO:0110089 |  |  |
|  | GO:0110091 |  |  |
|  | GO:0110090 |  |  |
|  | GO:0099072 |  |  |
|  | GO:0099601 |  |  |
|  | GO:0031547 |  |  |
|  | GO:1990790 |  |  |
|  | GO:0097150 |  |  |
|  | GO:0150011 |  |  |
|  | GO:1904391 |  |  |
|  | GO:0036489 |  |  |
|  | GO:0044467 |  |  |
|  | GO:0007272 |  |  |
|  | GO:0002087 |  |  |
|  | GO:0003070 |  |  |
|  | GO:0150012 |  |  |
|  | GO:0150013 |  |  |
|  | GO:0003118 |  |  |
|  | GO:0003119 |  |  |
|  | GO:0008065 |  |  |
|  | GO:0051394 |  |  |
|  | GO:0061772 |  |  |
|  | GO:0048755 |  |  |
|  | GO:0003090 |  |  |
|  | GO:1990089 |  |  |
|  | GO:0032455 |  |  |
|  | GO:0044487 |  |  |
|  | GO:0019226 |  |  |
|  | GO:0008088 |  |  |
|  | GO:0035082 |  |  |
|  | GO:0060404 |  |  |
|  | GO:0099612 |  |  |
|  | GO:0106092 |  |  |
|  | GO:0099124 |  |  |
|  | GO:0048678 |  |  |
|  | GO:0099003 |  |  |
|  | GO:0035418 |  |  |
|  | GO:0099643 |  |  |
|  | GO:0001771 |  |  |
|  | GO:0097120 |  |  |
|  | GO:0099179 |  |  |
|  | GO:0050808 |  |  |
|  | GO:0050803 |  |  |
|  | GO:2000574 |  |  |
|  | GO:0032598 |  |  |
|  | GO:0007268 |  |  |

**Supplementary File 4. Collection of GO terms grouped into different categories for multiple GO term testing.**
