# Supplementary material for: The Hox transcription factor Ubx stabilizes lineage commitment by suppressing cellular plasticity in Drosophila
Source: eLife. 2019 May 3;8:e42675. doi: 10.7554/eLife.42675 (PMC6513553; doi:10.7554/eLife.42675)
Supplement: Transparent reporting form [file elife-42675-transrepform.docx]

***eLife’s* transparent reporting form**

We encourage authors to provide detailed information *within their submission* to facilitate the interpretation and replication of experiments. Authors can upload supporting documentation to indicate the use of appropriate reporting guidelines for health-related research (see [EQUATOR Network](http://www.equator-network.org/%20)), life science research (see the [BioSharing Information Resource](https://biosharing.org/)), or the [ARRIVE guidelines](http://www.plosbiology.org/article/info:doi/10.1371/journal.pbio.1000412) for reporting work involving animal research. Where applicable, authors should refer to any relevant reporting standards documents in this form.

If you have any questions, please consult our Journal Policies and/or contact us: [editorial@elifesciences.org](mailto:editorial@elifesciences.org).

**Sample-size estimation**

- You should state whether an appropriate sample size was computed when the study was being designed
- You should state the statistical method of sample size computation and any required assumptions
- If no explicit power analysis was used, you should describe how you decided what sample (replicate) size (number) to use

Please outline where this information can be found within the submission (e.g., sections or figure legends), or explain why this information doesn’t apply to your submission:

Tissue-specific nuclei samples where isolated via the INTAC method (Materials and Methods). About 1 mio. Nuclei were used for genome-wide sequencing, the library was prepared by using the ThruPLEX DNA-Seq Kit (Rubicon). Single End sequencing resulted in 50 bp reads. (Materials and Methods, not described). Wildtype sequencing was performed in two replicated, because that was efficient and Ubx protein degradation in three replicated, because of the new functional data and to gain a robust data set.

**Replicates**

- You should report how often each experiment was performed
- You should include a definition of biological versus technical replication
- The data obtained should be provided and sufficient information should be provided to indicate the number of independent biological and/or technical replicates
- If you encountered any outliers, you should describe how these were handled
- Criteria for exclusion/inclusion of data should be clearly stated
- High-throughput sequence data should be uploaded before submission, with a private link for reviewers provided (these are available from both GEO and ArrayExpress)

Please outline where this information can be found within the submission (e.g., sections or figure legends), or explain why this information doesn’t apply to your submission:

Wildtype sequencing was performed in two biological replicated and Ubx protein degradation in three biological replicates. The biological replicated contained the same genotype and stages but were collected on a different day. The further processing was performed together to reduce any potential mistakes. The data will be uploaded on the GEO, our number for the full data set will be GSE121754, which contains already all the sets that were produced before the revision. Data sets of Pho-ChIP-seqs in the Loss of Ubx background will be added and uploaded.

Outliner: Ubx without any analyzed Histone mark, the date was not in the focus of the paper.

**Statistical reporting**

- Statistical analysis methods should be described and justified
- Raw data should be presented in figures whenever informative to do so (typically when N per group is less than 10)
- For each experiment, you should identify the statistical tests used, exact values of N, definitions of center, methods of multiple test correction, and dispersion and precision measures (e.g., mean, median, SD, SEM, confidence intervals; and, for the major substantive results, a measure of effect size (e.g., Pearson's r, Cohen's d)
- Report exact p-values wherever possible alongside the summary statistics and 95% confidence intervals. These should be reported for all key questions and not only when the p-value is less than 0.05.

Please outline where this information can be found within the submission (e.g., sections or figure legends), or explain why this information doesn’t apply to your submission:

For statistical analysis Students t-test, the wilcoxon rank test and fisher-test (p-values) for multiple testing of GO-terms was used. Genome wide experiments includes larger numbers of N. R script for mutable testing see below.

Embryonic staining and quantification:

Fig.3g,j: Ubx protein degradation in the mesoderm, x and in each embryo 5 segments, since Ubx is expressed from A1-A7 in the mesoderm, t-test: p-value 0.00203. dpp-lacZ in the Ubx protein degradation background, 6 embryos of the same stage were measured. t-test: p-value 0.014676

SubFif.4g: 5 embryos were measured, t-test: p-value: 3.97409E-08

Fig.4f: wilcoxon rank test: p-value: 0.054

Fig.6k,l: 5 embryos were measured and in each embryo 5 segments, since Ubx is expressed from A1-A7 in the mesoderm. Loss of Ptx in Ubx degrad: t-test:p-value: 0.00355, Loss of Ptx in pho&phoL mutant: t-test:p-value: 0.0081. Loss of vnd in Ubx degrad: t-test:p-value: 0.0268502, Loss of vnd in pho&phoL mutant: t-test:p-value: 0.00272

SubFig6e: 5 embryos were measured and in each embryo 5 segments. Loss of Ptx in Ubx degrad: t-test:p-value: 0.00156

SubFig7c: 5 embryos were measured and in each embryo 5 segments. Gain of Antp in Ubx degrad: t-test:p-value: 0.01464

(For large datasets, or papers with a very large number of statistical tests, you may upload a single table file with tests, Ns, etc., with reference to sections in the manuscript.)

**Group allocation**

- Indicate how samples were allocated into experimental groups (in the case of clinical studies, please specify allocation to treatment method); if randomization was used, please also state if restricted randomization was applied
- Indicate if masking was used during group allocation, data collection and/or data analysis

Please outline where this information can be found within the submission (e.g., sections or figure legends), or explain why this information doesn’t apply to your submission:

**Additional data files (“source data”)**

- We encourage you to upload relevant additional data files, such as numerical data that are represented as a graph in a figure, or as a summary table
- Where provided, these should be in the most useful format, and they can be uploaded as “Source data” files linked to a main figure or table
- Include model definition files including the full list of parameters used
- Include code used for data analysis (e.g., R, MatLab)
- Avoid stating that data files are “available upon request”

Please indicate the figures or tables for which source data files have been provided:

R script for multiple GO-term testing

R: multiple GO-term testing

library(GO.db)

library(biomaRt)

library(readr)

#-------------------------------------------------------------------------------

\# for creating a reduced list of non-overlapping, unique GO-terms

includesParent <- function(goid, all.goids) {

ont <- Ontology(goid)

anc <- c()

switch(ont,

BP = {

anc <- as.list(GOBPANCESTOR[goid])

},

MF = {

anc <- as.list(GOMFANCESTOR[goid])

},

CC = {

anc <- as.list(GOCCANCESTOR[goid])

})

return(any(unlist(anc) %in% all.goids))

}

reduceToParents <- function(goids) {

return(goids[!sapply(goids, includesParent, all.goids=goids)])

}

\# load list

\# then do:

\# goterms <- reduceToParents(goterms)

\# and write to file for further use

#-------------------------------------------------------------------------------

addOffspring <- function(goid) {

ont <- Ontology(goid)

off <- c()

switch(ont,

BP = {

off <- as.list(GOBPOFFSPRING[goid])

},

MF = {

off <- as.list(GOMFOFFSPRING[goid])

},

CC = {

off <- as.list(GOCCOFFSPRING[goid])

})

return(unique(c(goid, unlist(off))))

}

completeGOlist <- function(goids) {

goids <- unlist(sapply(goids, addOffspring))

goids <- unique(goids)

goids <- goids[complete.cases(goids)]

return(goids)

}

\# retrieve genes from biomart

mart <- useMart("ensembl", dataset = "dmelanogaster_gene_ensembl")

dmel <- getBM(c("flybase_gene_id", "go_id"), mart=mart)

dmel <- dmel[which(dmel$go_id != ""), ]

dmel <- dmel[which(dmel$flybase_gene_id != ""), ]

\# get sample file

message("input file for sample")

filename <- file.choose()

\# filter sample file

sample <- read_csv(filename, col_names = FALSE)

sample <- sample[[1]]

sample <- intersect(sample, dmel$flybase_gene_id)

\# get go terms to test

message("input file for GO terms")

filename <- file.choose()

goterms <- read_csv(filename, col_names = FALSE)

goterms <- completeGOlist(goterms[[1]])

\# calculate p Value with Fisher's exact test

genes.in.cat <- dmel[which(dmel$go_id %in% goterms), ]

n.tot <- length(unique(dmel$flybase_gene_id))

n.gic <- length(unique(genes.in.cat$flybase_gene_id))

n.gnic <- n.tot - n.gic

n.s <- length(unique(sample))

A <- length(unique(intersect(genes.in.cat$flybase_gene_id, sample)))

B <- n.s - A

C <- n.gic - A

D <- n.gnic - B

\# overrepresentation

res <- fisher.test(matrix(c(A,B,C,D), nrow=2, ncol=2), alternative="greater")

print(res)
